# Multi-biomarker is an early-stage predictor for progression of Coronavirus disease 2019 (COVID-19) infection

**DOI:** 10.7150/ijms.58742

**Published:** 2021-05-27

**Authors:** Zheng Zhou, Ying Li, Yuanhui Ma, Heng Zhang, Yunfeng Deng, Zuobin Zhu

**Affiliations:** 1 Katharine Hsu International Research Institute of Infectious Disease, Shandong Provincial Public Health Clinical Center, Shandong University, Jinan 250013, China.; 2 Medical Technology School of Xuzhou Medical University, Xuzhou 221004, China.; 3 Department of Pathology, Shandong Provincial Public Health Clinical Center, Shandong University, Jinan 250013, China.; 4 Department of Labor, Jining Psychiatric Hospital, Jining 272051, China.; 5 Department of Genetics, Xuzhou Medical University, Xuzhou 221004, China.

**Keywords:** COVID-19, predictors, Multi-biomarker, progression, IL-6

## Abstract

Coronavirus disease 2019 (COVID-19) has spread widely in the communities in many countries. Although most of the mild patients could be cured by their body's ability to self-heal, many patients quickly progressed to severe disease and had to undergo treatment in the intensive care unit (ICU). Thus, it is very important to effectively predict which patients with mild disease are more likely to progress to severe disease. A total of 72 patients hospitalized with COVID-19 in Shandong Provincial Public Health Clinical Center and 1141 patients included in the published papers were enrolled in this study. We determined that the combination of interleukin-6 (IL-6), Neutrophil (NEUT), and Natural Killer (NK) cells had the highest prediction accuracy (with 75% sensitivity and 95% specificity) for progression of COVID-19 infection. A binomial regression equation that accounted for a multiple risk score for the combination of IL-6, NEUT, and NK was also established. The multiple risk score is a good indicator for early stratification of mild patients into risk categories, which is very important for adjusting the treatment plan and preventing death.

## Introduction

Coronavirus disease 2019 (COVID-19), which is thought to be related to the severe acute respiratory syndrome (SARS), is triggered by SARS-COV2 and has become a public health emergency of international concern 1. COVID-19 is transmitted from human to human, mainly through droplet and contact routes. A wide range of signs, ranging from mild disease to severe symptoms, has been reported in patients with COVID-19 2,3. Generally, the current new coronavirus seems to have relatively low pathogenicity in mild patients but it may result in certain sequelae and high fatality rate among severe patients. Its R0 value can be as high as 5.7 4. Compared with influenza A in 2009 and Middle East Respiratory Syndrome (MERS) in 2014, COVID-19 is more infectious (i.e., its R0 value is greater). Until Oct 1, 2020, 34 million persons have been infected worldwide with the death toll topping 1,014,958.

At present, COVID-19 has spread widely in the communities in many countries 5. Because too many people have been infected, medical institutions have advised hospitalization for severe cases and home quarantine for mild cases. Although most of the mild patients could be cured by their body's ability to self-heal, many patients quickly progressed to severe disease and had to undergo treatment in the intensive care unit (ICU). Severe COVID-19 disease can cause great harm to the human body, has a high mortality rate, and may result in many sequelae, such as reduced pulmonary function and impaired nervous system function after treatment 6,7; therefore, it is important to effectively predict which patients with mild disease are more likely to progress to severe disease, in order to pay attention and provide early timely treatment.

Although some biomarkers, such as lymphocyte count, D-dimer, and interleukin (IL)-6, have been reported as risk factors for the severity of COVID-19 infection, most of these biomarkers can be used to distinguish patients with severe disease from normal persons or patients with mild disease 8; however, estimation of risk factors for COVID-19 disease progression in previous studies is not very robust. Since the risk of COVID-19 is affected by multiple biologically redundant factors, the relationships between these hematological biomarkers may contribute to predicting the progression of COVID-19. In many common diseases, polygenic risk scores of multi common variations provide better disease risk prediction than single rare or common mutations 9,10. A previous study has also shown that collective effects of common single nucleotide polymorphisms (SNPs), in which single variation has small effect size in diseases, could improve risk prediction of many diseases 11,12. A generalized linear model (GLM) is a good predictor that has feature importance measures and excellent predictive accuracy 13. In this study, the GLM was employed to determine the optimal combination of biomarkers for early prediction of the risk of patients with mild disease progressing to severe disease. As many countries slowly emerge from lockdown measures, early-stage predictors for progression of COVID-19 infection are of great value as early effective intervention can effectively protect the vulnerable population from COVID-19 and reduce the fear of disease, which is conducive to return to normal socially productive activities. Therefore, the primary aim of this study was to evaluate whether the multi-biomarker is a good early-stage predictor compared to a single biomarker for progression of COVID-19 infection.

## Materials and methods

### Data collection

This retrospective cohort study included 72 inpatients diagnosed with COVID-19 infection from January 29, 2020 to April 24, 2020 in the Shandong Provincial Public Health Clinical Center. The Research Ethics Commission of Shandong Provincial Public Health Clinical Center (2020XKYYEC-03) approved the study. Reverse transcription-polymerase chain reaction (RT-PCR) was used to confirm that all patients were positive for the new coronavirus nucleic acid. The World Health Organization (WHO) interim guidance for COVID-19 was used to diagnose the patients accordingly, and they were divided into mild and severe groups. Patients with mild disease met the following criteria: (1) RT-PCR positive result for SARS-COV2 RNA, (2) Fever or other respiratory signs, (3) Viral pneumonia abnormality diagnosed on a typical CT image. Patients with severe disease met at least one of the following criteria: (1) Shortness of breath, respiratory rate (RR) ≥ 30 breaths/min, (2) Oxygen saturation ≤ 93%, or (3) PaO2/FiO2 ≤ 300 mmHg. The WHO/International Severe Acute Respiratory and Emerging Infection Consortium case record form for severe acute respiratory infections was used to extract the epidemiological, demographic, clinical, laboratory, treatment, and outcome data from the electronic medical records.

### Laboratory procedures

Real-time PCR methods were used for determining the methods for laboratory validation of SARS-CoV-2 infection 2. After clinical remission of symptoms, including fever, cough, and dyspnea, throat swab specimens were collected for SARS-CoV-2 PCR retesting every other day; however, only qualitative data were available. Absence of fever for at least 3 days, substantial improvement in both lungs on chest CT, clinical remission of respiratory symptoms, and two throat swab samples negative for SARS-CoV-2 RNA obtained at least 24 h apart were the criteria for discharge. Routine hematological investigations were as follows: complete hematological count, coagulation profile, serum biochemical tests (including renal and liver function, creatine kinase, lactate dehydrogenase, and electrolytes), myocardial enzymes, and cytokines.

### Meta-analysis

#### Search strategy

This study was a review conducted in 2020. Searches were performed in the scientific PubMed database, using the combination of related keywords based of MeSH terms (Table 1). A researcher (Z. Z), a professional clinician, searched the PubMed database for all published articles on COVID-19 up to March 9, 2020 using the following keywords: “2019-nCoV”, “Coronavirus”, “COVID-19”, and “SARS-CoV-2”. Another researcher (L. Y), a professional clinician with expertise in systematic reviews, independently repeated the first reviewer's search. Both searches were in complete agreement with each other. All steps of searches were performed based on the Preferred Reporting Items for Systematic Reviews and Meta-Analyses (PRISMA) checklist. Searches were limited to papers published in English and Chinese languages.

#### Inclusion and exclusion criteria

The inclusion criteria were as follows: original articles, retrospective case series, and case reports of COVID-19 infection, including clinical features, epidemiological findings, laboratory and imageological examination, treatment options, or pathological studies. Exclusion criteria were as follows: non-availability of full text, no target observations, and other article types. Other article types included review articles, comments, and news. Data extraction included that of Lymphocytes, CD3+ T cells, Neutrophils (NEUT), Platelet count (PLT), CD4+ T cells, CD8+ T cells, C-reactive protein (CRP), D-dimer, Natural Killer (NK) cells, White blood cell count (WBC), Fibrin degradation products (FDP), Thrombin time (TT), Activated partial thromboplastin time (APTT), IL-10, IL-6, and Platelet distribution width (PDW). After data extraction, the findings were summarized and reported in tables and figures according to the objectives of the study. Two researchers (Z. Z and L. Y who were specialist physicians) reviewed all articles in detail. The researchers identified all articles presenting the clinical characteristics or pathologic studies of COVID-19 infection. The search results were submitted to a third party (M. Y. H who was a professionally trained physician), which reviewed the discrepancies and made decisions in the event of disagreement (Table 2).

#### Study characteristics and quality assessment

The 18 included studies were observational studies. A total of 16 studies were from China, and they included more than 18 provinces and cities. The time span of the study period was from 11^th^ November 2019 to 23^rd^ September 2020. With respect to the comparison of mild and severe patients, 16 studies 21, 22-37 described patient characteristics, 9 studies described comorbidities 21, 24-27, 29-32, 4 studies 34, 35, 36, 37, 38 described vital signs, 7 studies compared symptoms 21-27, and 18 studies 21-38 described laboratory findings.

We appraised the trial quality using the Cochrane collaboration tool for assessing the risk of bias (ROB), including assessment of random sequence generation, allocation concealment, blinding (of interventions and outcome measurement or assessment), incomplete outcome data, selective reporting bias, and other potential sources of bias (e.g., age). For each criterion, we appraised the ROB as being either low, high, or unclear risk (e.g., insufficient details). Two authors (Z.Z and L Y) independently assessed the study quality and disagreements were resolved by consensus.

#### Data synthesis and meta-analysis

For continuous outcomes, standardized mean difference (SMD) with the corresponding 95% CI was calculated. Cochran Chi-square test and I^2^ were used to assess the heterogeneity among studies. A fixed-effects model was used when I^2^ was < 50%, while a random-effects model was selected when I^2^ was > 50%. If there was statistical heterogeneity among the results, further sensitivity analysis was conducted to determine the source of heterogeneity. After significant clinical heterogeneity was excluded, the randomized effects model was used for meta-analysis. Publication bias was evaluated using Egger's test (Table 3). P < 0.05 was considered to indicate statistical significance. All data were analyzed using the Review Manager 5.2 software.

### Statistical analysis

The means and standard deviations were used to represent continuous variables. Percentages were used to represent categorical variables. The biomarkers, which showed differences between the patients with mild disease and severe disease, were examined by the Mann Whitney test. The incidence of clinical disease, which differed between mild and severe patients, was examined by Fisher's exact test. The sensitivity (true positive rate, TPR) and specificity (true negative rate, TNR) were then calculated using Prism 5 12. GLMs and Pearson correlation test were performed on R-Studio version 1.2.5033. GLM covariates were selected using binomial regression and the best fit subset using the Bayesian information criterion (BIC).

## Results

### Baseline patient and disease characteristics

The study population included 72 hospitalized patients diagnosed with COVID-19 in Shandong Provincial Public Health Clinical Center before April 24, 2020. Among the 72 patients, 56 were categorized as having mild disease, and 16 were categorized as having severe disease. Patients in the severe disease group (n = 16) were significantly older (median age, 60 years vs. 47 years; p < 0.05) and were more likely to have clinical comorbidities, including hypertension (50.00% vs. 14.30%), diabetes (25.00% vs. 10.2%), coronary heart disease (25% vs. 6.1%), and cerebrovascular disease (31.3% vs. 6.1%) when compared with patients in the mild disease group (n = 56) (Table 4).

### Hematological biomarkers could distinguish between mild and severe patients

Hematological biomarkers, including total hematological count, agglutination profile, serum biochemical tests, myocardial enzymes, lymphocyte subsets, and cytokine profiles, were examined. Twenty-eight hematological biomarkers showed a significant difference between mild and severe patients (Table 5). Then the true positive rate (TPR) of each hematological biomarker was calculated. Sixteen hematological biomarkers that showed good discriminatory capability were finally identified (P < 0.05, TPR > 30%) (Table 5 and Supplemental Figure 1).

Then, the discriminatory capability of 16 hematological biomarkers was confirmed by systematic review of the data published all over the world. Finally, a total of 18 articles out of the 178 articles that were retrieved, were included in the meta-analysis, which comprised data from 1141 patients, after excluding the following papers: 85 papers were excluded due to repeated retrieval, 46 papers were excluded after reading the abstracts, and 29 papers were excluded after reading the full text. Through the meta-analysis, it was found that most of these hematological biomarkers could effectively distinguish patients with mild disease from patients with severe disease.

### None of the single hematological biomarkers could effectively predict disease progression in patients with mild disease

**Figure 1:**
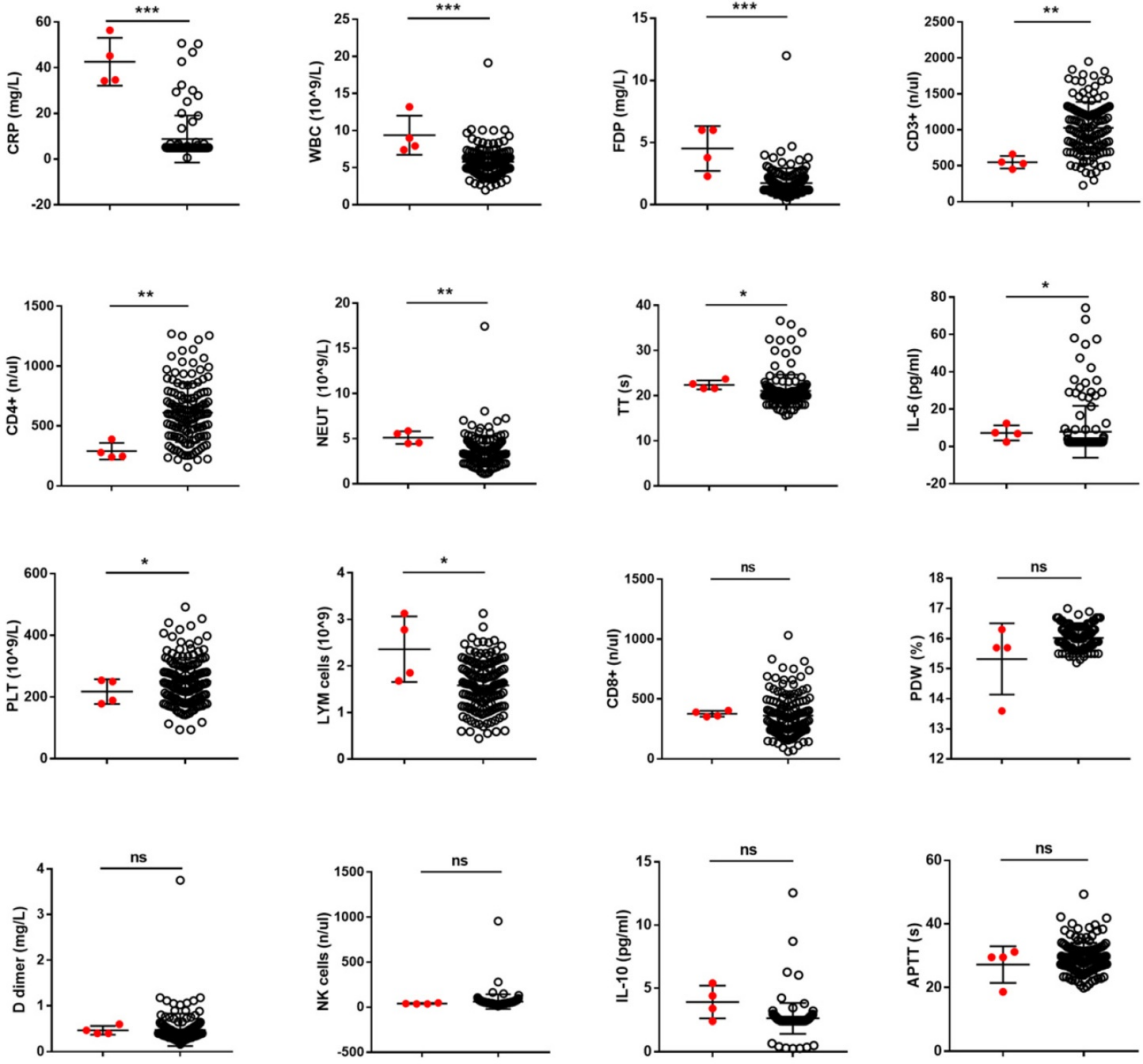
** The value of hematological biomarkers in predicting the progression of COVID-19**. Hematological biomarkers can be better predictors if we can use them to identify the patients with poor prognosis from the population with good prognosis. In this study, the predictive ability of 16 hematological biomarkers for COVID-19 infection progression, which showed a significant difference between mild patients and severe patients, confirmed in our data and in a systematic review was further assessed.

During hospitalization of 72 patients, most of the hematological biomarkers were detected more than 3 times. Among these 72 patients, 4 patients had complete data from mild to severe disease status. We used these 4 patients to examine the prediction effect of the 16 biomarkers and the value of 10 biomarkers, especially CRP, WBC, and FDP showed a significant difference between these 4 patients in a mild state with poor prognosis and mild patients with good prognosis (P <0.05). However, none of the single biomarkers could effectively predict the progression of COVID-19 (Fig. 1).

### Multiple-factor risk score had a better prediction effect for progression of COVID-19 than single hematological biomarkers

Hematological biomarkers, including complete hematological count, serum biochemical tests, coagulation profile, myocardial enzymes, lymphocyte subsets, and cytokine profiles, were examined using the venous blood obtained simultaneously. Thus, the combined effect of different biomarkers on COVID-19 infection could be analyzed. Here, combinations of biomarkers were first studied with respect to whether they could be used as early-stage predictive markers for progression of COVID-19 infection.

**Figure 2:**
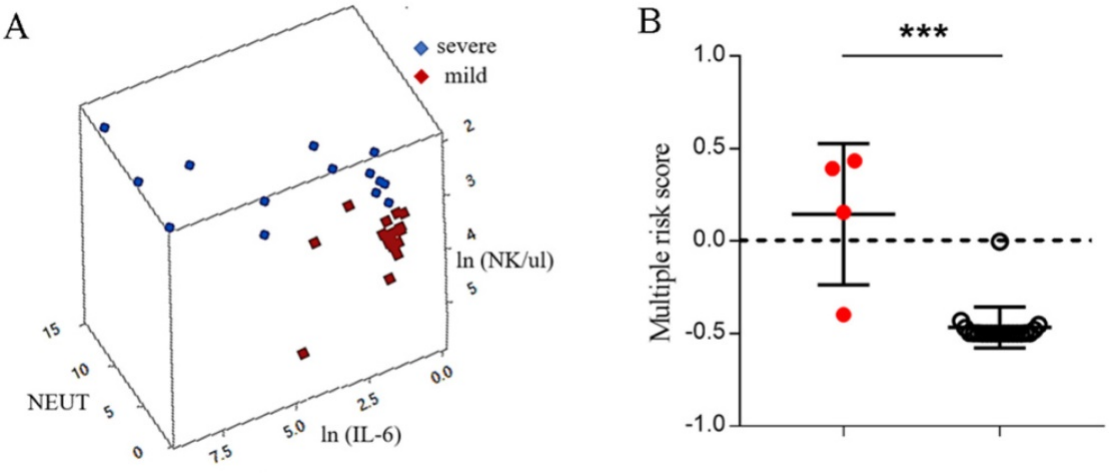
** Multiple risk score has a better prediction effect for the progression of COVID-19**. (A) The ability of the combination of IL-6, neutrophil granulocytes, and NK cells to distinguish between mild and severe patients. (B) The value of the combination of IL-6, neutrophil granulocytes, and NK cells in predicting the progression of COVID-19 by using an independent dataset. If the multiple risk score is greater than 0, the mild patient has a high probability of progressing to severe disease; if the multiple risk score is less than 0, the mild patient has a low probability of progressing to severe disease.

A GLM was used to analyze the interaction between the 19 biomarkers. Sixteen biomarkers were incorporated into the GLM as variables. The optimal models were three-dimensional models with the highest discrimination capability of 94.12% (P < 0.001) (Fig. 2A). These results indicated that the IL-6, neutrophil granulocytes, and NK cells exhibited interaction effects on COVID-19 infection. A binomial regression equation was then presented by using IL-6, neutrophil granulocytes, and NK cells to calculate the multiple-factor risk score for progression and survival of COVID-19. The binomial regression equation was -(exp ( -30.140 -1.821 × NEUT + 10.519 × ln (NK/ul) + 0.305 × ln(IL-6)) / (1+exp (-30.140 -1.821 × NEUT +10.519 ×ln (NK/ul) + 0.305 × ln(IL-6))-0.5). The value of IL-6, NEUT cells, and NK cells in patients with mild disease was imported into the binomial regression equation; if the score was greater than 0, the patient with mild disease had a high probability of progressing to severe disease; and if the score was less than 0, the patient with mild disease had a low probability of progressing to severe disease. By using the binomial regression equation, it was found that the combination of biomarkers, including IL-6, neutrophil granulocytes, and NK cells, showed a better discriminating ability than the optimal single biomarker (75% vs 25%) (Fig. 2B).

## Discussion

WHO recommends that all patients with new coronavirus pneumonia should be kept under observation in medical institutions. When it is impossible to keep the patients under observation in a medical institution due to objective reasons, home isolation and observation are also a viable strategy 14. In many countries, only people with severe symptoms are tested for new coronavirus and treated in hospitals. Most of the people with mild symptoms are recommended home quarantine and are sent to a hospital for treatment if their condition becomes serious. Most of the patients with mild disease can be cured by their body's ability to self-heal, but some of them deteriorate quickly; and once they develop severe disease, it would cause great harm to their body and may result in sequelae. The formation of scars results in decreased lung capacity. There is no long-term follow-up investigation for severe COVID-19 patients after recovery, but from 2003 to 2018, 71 SARS patients were followed up; it was found that more than one-third of patients had residual scars in their lungs 15. Among the 36 surviving MERS patients, about one-third of the patients also had long-term lung injury 16. In addition, the scarring rate in patients with COVID-19 may eventually be higher than that in patients with SARS and MERS because these diseases usually affect only one lung and COVID-19 frequently seems to affect both lungs, which also exacerbates the risk of lung scarring 17. Impaired lung function caused by SARS-COV-2 infection could negatively affect other organs (such as heart, kidney, and brain), and health effects of this infection may persist after the disease is cured.

According to the WHO report, about 10 to 15% of patients with mild and moderate disease will develop severe disease and the disease course in some patients is rapid 18. Evaluation of the risk of developing severe disease among mild patients, isolation of low-risk patients at home, and treatment of high-risk mild patients in medical institutions in a timely manner can not only reduce the burden on medical resources, but can also effectively reduce the proportion of severe patients as well as the mortality.

Early identification and management of mild patients are essential to reduce the incidence of severe disease. It has been reported that many biomarkers showed a significant difference between mild and severe patients 17,18. A systematic review and meta-analysis suggested that elevated procalcitonin, CRP, D-dimer, and LDH and decreased albumin can be used for predicting severe outcomes in COVID-19 19. Our data also confirmed some of the hematologic markers. Furthermore, in order to make early predictions, the predictors presented in our study were obtained by comparing the hematologic markers between severe patients with mild symptoms and patients with mild symptoms who did not eventually develop severe symptoms. Sixteen biomarkers were selected, which showed a significant difference between mild and severe patients in Shandong Provincial Public Health Clinical Center and were confirmed by systematic review of 18 published articles. In medical practice, sensitivity (TPR) and specificity (TNR) are often used to assess the accuracy and effectiveness of a biomarker in disease prediction. In this study, none of the 16 biomarkers showed good sensitivity and specificity in the training data. Herein, hematological biomarkers at different time points were recorded in four mild patients whose symptoms worsened rapidly and became severe, which could be used to further verify the prediction effect of these biomarkers. On comparing the two groups of 4 patients with poor prognosis and mild patients with good prognosis, 10 biomarkers showed statistically significant differences (P <0.05); however, it was not possible to distinguish these 4 patients with poor prognosis from the population with good prognosis based on any single biomarker. This result suggests that although most of the biomarkers could distinguish between mild and severe disease, the ability to predict the progression of COVID-19 infection was insufficient.

Previous work has shown that multiple variations in which a single SNP has small effect size can improve risk prediction of many diseases 11,12. Currently, there is no standard method to analyze and interpret the data of multiple biomarkers. A method known as GLM, which is conventionally used to understand genetic epistasis, was first used to identify biomarker relationships. Contrary to genetic markers that are immobile, hematological biomarkers are mobile. Therefore, in order to truly evaluate the correlation between different biomarkers, all blood samples were collected on the same day and at the same time, rather than performing different biomarker tests at different time points.

A good predictive model should not be disturbed by clinical factors to a large extent. The biomarkers, such as IL-6, NEUT, and NK cells, in this study were selected from the clinical routine test index, and some important potential confounders, such as age and the basic disease; therefore, age and clinical symptoms had minimal influence on the predicted results. This study determined that the IL-6, NEUT, and NK cell combination, which showed good prediction of COVID-19, had 93% sensitivity and 100% specificity in the training data. In the independent test data, the IL-6, NEUT, and NK cell combination had a good predictive value with 75% sensitivity and 95% specificity. Among these indicators, IL-6 is a good predictor and an effective target for drug therapy 20. Our results suggested that the combination of IL-6, NEUT, and NK cells had a good predictive ability than a single biomarker for progression of COVID-19 infection. Furthermore, the results of this study revealed that the combination of IL-6, NEUT, and NK cells had a good discriminating ability.

This study has some limitations. The study was limited by the number of patients who had complete data of hematological biomarkers, from mild status to severe status, because many patients had a very severe status on admission, and the combination of primary and secondary data in this study could have resulted in multiple biases. Therefore, the interacting biomarkers identified in this study need to be validated further in more mild patients with different outcomes and more samples from different countries or regions. Evaluation of these biomarkers in a longitudinal study is another way to address this limitation.

## Supplementary Material

Supplementary figure.

## Figures and Tables

**Table 1:** Search strategy for the research

	Search strategy
Database	PubMed
Limitations	Language (English or Chinese), Species (studies in humans)
Data	2019 to March 9, 2020
#1 (MeSH)	“COVID-19 virus” and “Cytokines”
#2 (Entry Terms)	“COVID 19 virus” or “COVID-19 virus” or “coronavirus disease” or “2019 virus” and “Cytokines”
Search	#1 or #2

**Table 2:**
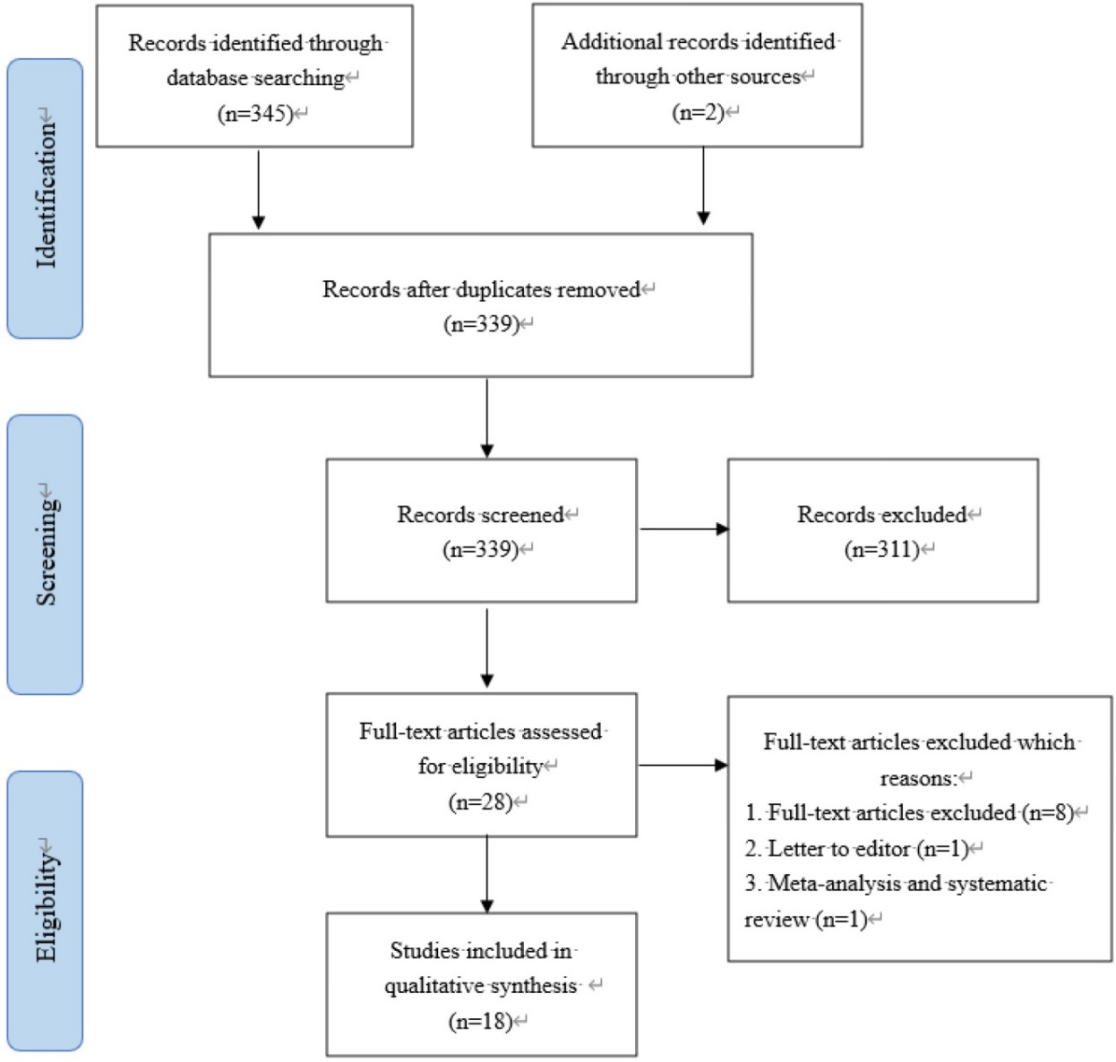
Search strategy used in the meta-analysis for selecting patients for inclusion in the study

**Table 3:** Meta-analysis of 18 studies reporting the association between 16 hematological biomarkers and severity of COVID-19 infection

Biomarkers	N of Study	SMD (95%CI)	I^2^	Chi-square (P-value)	Egger test (P-value)
Lymphocyte (**×**10^9^/L)	9 [21,22,23,25,26.27,28,29,31]	1.48 (1.30; 1.67)	97.0	289.26 (P < 0.001)	0.69 (P = 0.51)
CD3+ T cell (**×**10^12^/L)	8 21,23,25,27,28,29,30,33	1.28 (1.09; 1.47)	94	108.54 (P < 0.001)	1.69 (P = 0.23)
Neutrophils (**×**10^9^/L)	9 21,22,23,25,26,27,28,29,31	-2.06 (-2.26; -1.86)	96	178.38 (P < 0.001)	0.01 (P = 0.99)
PLT (**×**10^9^/L)	2 23,25,27	0.37 (0.05; 0.69)	0.0	1.81 (P=0.40)	0.09 (P = 0.94)
CD4+ T cell (**×**10^12^/L)	8 21,23,25,27,28,29,30,33	2.11 (1.91; 2.31)	82	39.37 (P < 0.001)	2.84 (P = 0.04)
CD8+ T cell (**×**10^12^/L)	8 21,23,25,27,28,29,30,33	1.00(0.83; 1.18)	88	58.60 (P < 0.001)	1.82 (P = 0.11)
CRP (mg/L)	9 22,23,24,25,27,28,31,33,35	-0.83 (-1.07; -0.58)	96	226.59 (P < 0.001)	1.2 (P = 0.36)
DD-dimer (μg/mL)	4 23,25,26,28	-1.75 (-1.99; -1.50)	98	122.56 (P < 0.001)	1.37 (P = 0.3)
NK cell (**×**10^6^/L)	3 21,25,30	21.21 (18.28; 24.15)	56	4.50 (P < 0.001)	0.81 (P = 0.46)
WBC (×10^9^/L)	8 21,22,27,28,29,31,32,36	-1.30 (-.1.54; -1.07)	95	141.24 (P < 0.001)	0.42 (P = 0.05)
FDP (mg/L)	4 27,36,37,38	-1.19 (-1.57; -0.81)	94	52.53 (P < 0.001)	2.02 (P = 0.09)
TT (s)	4 27,36,37,38	0.30 (-0.03; 0.64)	78	13.61 (P < 0.001)	1.06 (P = 0.32)
APTT (s)	4 27,36,37,38	-0.26 (-0.63; 0.10)	94	51.80 (P < 0.001)	0.37 (P = 0.73)
IL-10 (pg/ml)	8 21,23,25,27,28,29,30,31	-1.58 (-1.86; -1.31)	97	221.08 (P < 0.001)	1.66 (P = 0.34)
IL-6 (pg/ml)	15 21,22,23,24,25,26,27,28,29,30,31,32,33,34,35	-6.08 (-6.41; -5.75)	95	298.64 (P < 0.001)	0.91 (P = 0.53)
PDW (%)	4 22,31,37,38	0.43 (0.10; 0.75)	94	47.26 (P < 0.001)	0.67 (P = 0.31)

N: number of studies used. SMD: Standard Mean Difference. I^2^ was used for quantifying inconsistency: the larger the value, the stronger the heterogeneity.

**Table 4:** The clinical characteristics of COVID-19 patients

Characteristics	Mild (n=56)	Severe (n=16)	^5^P value
^1^Age (Mean ± SD)	47±16.44	60±16.88	0.0153
**Subgroup (%)**			
≤65 years old	87.50	62.50	
>65 years old	12.50	37.50	
**^2^Coexisting conditions (%)**			
Hypertension	14.30	50.00	0.009262
Diabetes	10.20	25.00	ns
Coronary heart disease	6.10	25.00	ns
Cerebrovascular disease	6.10	31.30	0.026548
Chronic obstructive pulmonary disease	1.80	6.30	ns
Chronic renal disease	0	6.30	ns
Cancer	5.40	6.30	ns
Tuberculosis	1.80	0	ns
**^3^Current smoker (%)**	17.90	16.67	ns
**^4^Positive culture on the same day plasma collected**			
Bacterial (%)	5.40	25.00	ns
Fungal (%)	1.80	12.50	ns

Note: ^1^ Age: the mean age of 56 mild patients and 16 severe patients. ^2^ Coexisting conditions (%): The percentage of coexisting diseases in mild and severe cases. Coexisting conditions were recorded in 49 mild patients and 16 severe patients. ^3^ Current smoker (%): The percentage of smokers in 49 mild patients and 16 severe patients. ^4^ Positive culture on the same day plasma: The percentage of fungi and bacteria detected in plasma of mild and severe patients. ^5^ P value: The incidence of clinical disease, which was different between mild and severe patients, was examined by Fisher's exact test.

**Table 5:** The laboratory characteristics between mild COVID-19 patients and severe COVID-19 patients

Biomarkers	Mild patients			Severe patients			P-value^5^	TPR^6^	TNR^7^
	Median^1^	Min^2^, Max^3^	Std. D^4^	Median^1^	Min^2^, Max^3^	Std. D^4^			
LYM cells (10^9^)	1.71	(0.76, 2.47)	0.53	0.68	(0.26, 1.53)	0.42	3.30E-06	0.23	0.80
CD3+ (n/ul)	1185.00	(405.1, 1840)	477.30	401.90	(104.3, 922)	281.30	2.90E-05	0.23	0.60
NEUT (10^9^/L)	3.33	(1.57, 5.34)	0.94	6.01	(1.85, 13.36)	3.30	9.50E-05	0.13	0.25
PLT (10^9^/L)	243.50	(178, 398)	50.87	151.50	(73, 310)	75.72	0.00029	0.21	0.16
CD4+ (n/ul)	637.50	(235.6, 1270)	343.90	270.30	(52, 657.6)	203.70	0.00030	0.38	0.43
CD8+ (n/ul)	379.20	(142.9, 1032)	222.10	127.50	(44.72, 365)	91.72	0.00036	0.15	0.65
CRP (mg/L)	5.00	(<5, 50.7)	10.79	19.97	(5, 124.8)	36.48	0.0008	0.15	0.13
D dimer (mg/L)	0.41	(0.19, 1.02)	0.21	1.64	(0.26, 7.86)	2.52	0.00212	0.13	0.12
NK cells (n/ul)	61.28	(28.55, 278.9)	55.33	19.49	(7.74, 42.53)	11.27	0.00257	0.15	0.15
WBC (10^9^/L)	5.50	(2.99, 8.38)	1.33	7.53	(2.96, 13.93)	3.14	0.00412	0.13	0.22
FDP (mg/L)	1.60	(0.9, 3.8)	0.77	5.80	(1.4, 34.1)	10.36	0.00414	0.17	0.26
TT (s)	21.00	(18.1, 23.2)	1.25	20.15	(17.4, 21)	1.58	0.00470	0.13	0.14
APTT (s)	26.95	(19.9, 33.3)	3.90	31.00	(23.4, 51.7)	8.51	0.00662	0.21	0.21
IL-10 (pg/ml)	2.44	(2.44, 8.72)	1.39	4.71	(2.44, 33.32)	8.68	0.02014	0.15	0.58
IL-6 (pg/ml)	2.44	(2.44, 58.1)	14.11	15.91	(2.44, 6040)	2144.00	0.02591	0.38	0.05
PDW (%)	15.80	(15.5, 16.5)	0.33	16.20	(15.2, 17.5)	0.82	0.02705	0.13	0.12
MCHC (pg)	325.50	(314, 341)	7.22	318.00	(283, 334)	14.38	0.00044	0.00	0.07
RDW-SD (%)	37.00	(31.9, 38.8)	1.98	45.15	(36.9, 84.4)	14.01	0.00044	0.00	0.04
PCT (%)	0.21	(0.15, 0.33)	0.04	0.14	(0.08, 0.31)	0.07	0.00076	0.00	0.09
INR	0.96	(0.84, 1.12)	0.08	1.14	(0.87, 1.74)	0.22	0.00088	0.04	0.08
PT (s)	11.10	(9.8, 13)	0.98	13.15	(10.1, 20.8)	2.65	0.00109	0.04	0.05
RBC (10^12^/L)	4.20	(3.02, 4.77)	0.52	3.10	(2.26, 5.16)	0.83	0.00223	0.04	0.04
Hb (g/L)	118.50	(92, 141)	13.03	94.50	(62, 150)	24.39	0.00487	0.04	0.09
ESR (mm/h)	15.00	(6, 102)	22.48	57.00	(6, 140)	46.21	0.00510	0.00	0.04
MCV (fl)	91.30	(76.6, 96.8)	5.84	95.65	(84.4, 114.5)	8.99	0.00773	0.04	0.05
Hct (%)	37.00	(26.9, 42.3)	3.89	29.60	(21.8, 47.1)	6.91	0.01654	0.04	0.05
RDW-CV (%)	12.75	(11.2, 15.3)	1.08	13.95	(12.7, 31.2)	5.86	0.01668	0.04	0.17
TNF-a (pg/ml)	13.82	(2.44, 138.6)	41.48	4.85	(2.44, 12.1)	3.01	0.03608	0.00	0.20

1 Median: The intermediate value of each biomarker. 2 Min: The minimum value of each biomarker. 3 Max: The maximum value of each biomarker. 4 Std. D: The standard deviation of each biomarker. 5 P-value: P-value were calculated by Mann Whitney test. 6 TPR: The true positive rate. 7 TNR: The true negative rate.
