# Supplementary material for: Multi-biomarker is an early-stage predictor for progression of Coronavirus disease 2019 (COVID-19) infection
Source: Int J Med Sci. 2021 May 27;18(13):2789–98. doi: 10.7150/ijms.58742 (PMC8241766; doi:10.7150/ijms.58742)

Supplementary Figure 1: Meta-analysis of the risk factors between mild patients and severe patients.

	FDP-Mild			FDP-Severe				Std. Mean Difference
Study or Subgroup	Mean	SD	Total	Mean	SD	Total	Weight	IV, Fixed, 95% CI
Han Y 2020	3.84	0.21	23	5.32	0.56	24	17.0%	-3.41 [-4.33, -2.49]
Liu J 2020	4.89	1.23	27	4.39	0.98	13	32.2%	0.42 [-0.25, 1.09]
Yang Y 2020	3.23	0.78	22	3.89	0.67	14	29.1%	-0.87 [-1.58, -0.17]
Zhang HZ 2020	2.85	0.67	29	5.01	1.32	14	21.7%	-2.29 [-3.10, -1.47]
**Total (95% CI)**			**101**			**65**	**100.0%**	**-1.19 [-1.57, -0.81]**

Heterogeneity: Chi² = 52.53, df = 3 (P < 0.00001); I² = 94%
Test for overall effect: Z = 6.17 (P < 0.00001)

Std. Mean Difference
IV, Fixed, 95% CI

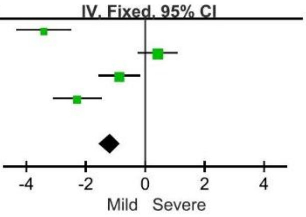

	TT-Mild			TT-Severe				Std. Mean Difference
Study or Subgroup	Mean	SD	Total	Mean	SD	Total	Weight	IV, Fixed, 95% CI
Han Y 2020	18.3	1.23	29	16.3	2.1	14	23.2%	1.26 [0.56, 1.96]
Liu J 2020	17.2	2.4	27	17.2	3.4	13	25.7%	0.00 [-0.66, 0.66]
Yang Y 2020	15.9	3.2	22	17.3	1.9	14	24.3%	-0.49 [-1.17, 0.19]
Zhang HZ 2020	20.1	4.3	29	18.2	2.5	14	26.9%	0.49 [-0.16, 1.13]
**Total (95% CI)**			**107**			**55**	**100.0%**	**0.30 [-0.03, 0.64]**

Heterogeneity: Chi² = 13.61, df = 3 (P = 0.003); I² = 78%
Test for overall effect: Z = 1.77 (P = 0.08)

Std. Mean Difference
IV, Fixed, 95% CI

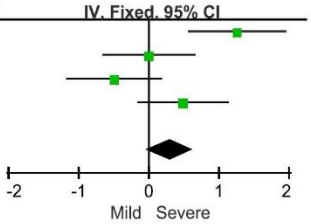

	APTT-Mild			APTT-Severe				Std. Mean Difference
Study or Subgroup	Mean	SD	Total	Mean	SD	Total	Weight	IV, Fixed, 95% CI
Han Y 2020	38.3	3.36	29	36.7	1.1	14	31.3%	0.55 [-0.10, 1.20]
Liu J 2020	36.7	5.4	27	37.2	2.4	13	30.1%	-0.11 [-0.77, 0.56]
Yang Y 2020	31.2	1.2	22	39.3	1.9	14	6.3%	-5.26 [-6.71, -3.81]
Zhang HZ 2020	37.1	4.3	29	38.2	5.5	14	32.3%	-0.23 [-0.87, 0.41]
**Total (95% CI)**			**107**			**55**	**100.0%**	**-0.26 [-0.63, 0.10]**

Heterogeneity: Chi² = 51.80, df = 3 (P < 0.00001); I² = 94%
Test for overall effect: Z = 1.42 (P = 0.16)

Std. Mean Difference
IV, Fixed, 95% CI

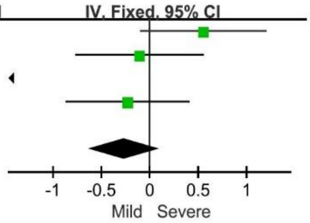

	IL-10-Mild			IL-10-Severe				Std. Mean Difference
Study or Subgroup	Mean	SD	Total	Mean	SD	Total	Weight	IV, Fixed, 95% CI
Chen G 2020	2.44	0.02	10	4.84	1.23	11	5.2%	-2.58 [-3.80, -1.36]
Di Q 2020	2.13	0.05	51	3.98	0.34	45	5.4%	-7.79 [-8.99, -6.60]
Fu S 2020	2.05	0.78	21	5.34	0.21	29	4.1%	-6.13 [-7.50, -4.76]
Nie SK 2020	2.9	0.23	72	3.01	0.11	25	35.9%	-0.53 [-0.99, -0.07]
Wan SX 2020	2.78	0.54	45	2.98	0.09	18	25.1%	-0.43 [-0.98, 0.12]
Wang WJ 2020	2.54	0.72	10	3.78	1.21	11	8.6%	-1.18 [-2.12, -0.24]
Zhang BC 2020	2.87	0.09	16	4.01	1.11	16	12.4%	-1.41 [-2.20, -0.63]
Zhang HZ 2020	2.14	0.17	29	3.99	0.47	14	3.5%	-6.06 [-7.55, -4.57]
**Total (95% CI)**			**254**			**169**	**100.0%**	**-1.58 [-1.86, -1.31]**

Heterogeneity: Chi² = 221.08, df = 7 (P < 0.00001); I² = 97%
Test for overall effect: Z = 11.22 (P < 0.00001)

Std. Mean Difference
IV, Fixed, 95% CI

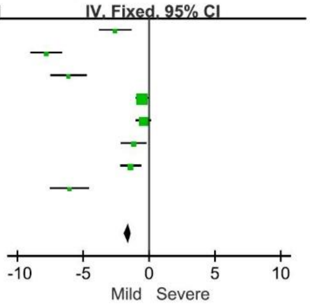

	PDW-Mild			PDW-Severe				Std. Mean Difference
Study or Subgroup	Mean	SD	Total	Mean	SD	Total	Weight	IV, Fixed, 95% CI
Feng C 2020	14.2	2.2	106	13.43	2.8	26	55.8%	0.33 [-0.10, 0.76]
Han Y 2020	10.9	0.67	29	9.43	0.78	14	16.9%	2.04 [1.26, 2.82]
Liu J 2020	9.3	1.98	27	8.9	0.52	13	23.5%	0.24 [-0.43, 0.90]
Wang WJ 2020	7.9	0.67	10	12.35	1.23	11	3.7%	-4.25 [-5.92, -2.59]
**Total (95% CI)**			**172**			**64**	**100.0%**	**0.43 [0.10, 0.75]**

Heterogeneity: Chi² = 47.26, df = 3 (P < 0.00001); I² = 94%
Test for overall effect: Z = 2.59 (P = 0.010)

Std. Mean Difference
IV, Fixed, 95% CI

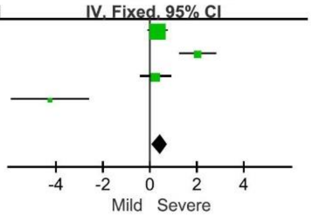

Study or Subgroup	CD8+ T cell-Mild Mean	SD	Total	CD8+ T cell-Severe Mean	SD	Total	Weight	Std. Mean Difference IV, Fixed, 95% CI
Chen G 2020	456	110	10	289	34	11	2.6%	2.01 [0.92, 3.10]
Di Q 2020	329	45	51	298	67	45	18.3%	0.55 [0.14, 0.95]
Fu S 2020	589	109	21	393	134	29	7.4%	1.55 [0.91, 2.20]
Nie SK 2020	561	98	72	498	123	25	14.3%	0.60 [0.13, 1.06]
Wang WJ 2020	673	209	10	489	198	11	3.7%	0.87 [-0.04, 1.77]
Yang G 2020	474	49	125	390	98	126	43.5%	1.08 [0.81, 1.34]
Zhang BC 2020	594	34	16	213	32	16	0.3%	11.25 [8.23, 14.27]
Zhang HZ 2020	430	13	29	412	18	29	9.9%	1.13 [0.57, 1.69]
**Total (95% CI)**			**334**			**292**	**100.0%**	**1.00 [0.83, 1.18]**

Heterogeneity: Chi² = 58.60, df = 7 (P < 0.00001); I² = 88%
Test for overall effect: Z = 11.24 (P < 0.00001)

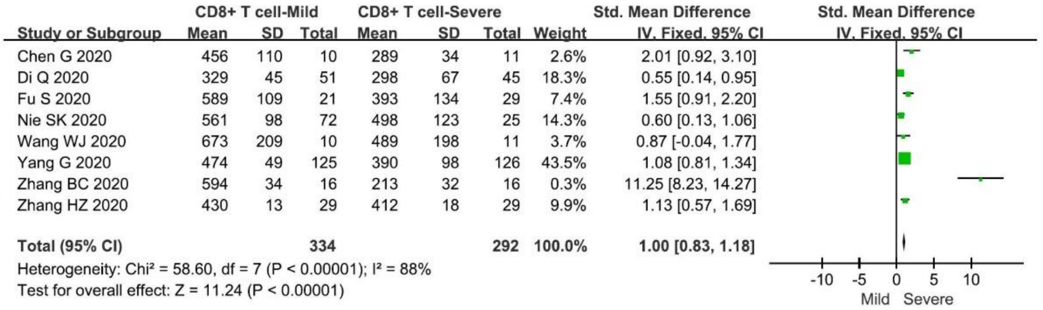

Study or Subgroup	CRP-Mild Mean	SD	Total	CRP-Severe Mean	SD	Total	Weight	Std. Mean Difference IV, Fixed, 95% CI
Chen G 2020	6.78	0.12	10	12.22	0.34	11	0.1%	-20.08 [-26.87, -13.29]
Chen XP 2020	4.98	1.23	14	7.89	0.27	11	4.2%	-2.99 [-4.19, -1.79]
Di Q 2020	8.67	0.56	51	8.34	0.23	45	34.8%	0.75 [0.33, 1.16]
Feng C 2020	7.45	0.39	106	8.9	2.09	26	27.7%	-1.47 [-1.93, -1.00]
Fu S 2020	4.89	0.34	21	9.83	0.49	29	1.1%	-11.21 [-13.56, -8.85]
Giuseppe G 2020	6.09	1.27	16	6.9	0.87	5	5.7%	-0.65 [-1.68, 0.38]
Wang WJ 2020	7.9	1.2	10	8.33	0.9	11	8.0%	-0.39 [-1.26, 0.47]
Wang Y 2020	7.32	0.34	26	8.98	2.23	20	15.2%	-1.10 [-1.73, -0.47]
Zhang HZ 2020	5.98	0.04	29	8.45	0.78	14	3.2%	-5.50 [-6.88, -4.13]
**Total (95% CI)**			**283**			**172**	**100.0%**	**-0.83 [-1.07, -0.58]**

Heterogeneity: Chi² = 226.59, df = 8 (P < 0.00001); I² = 96%
Test for overall effect: Z = 6.63 (P < 0.00001)

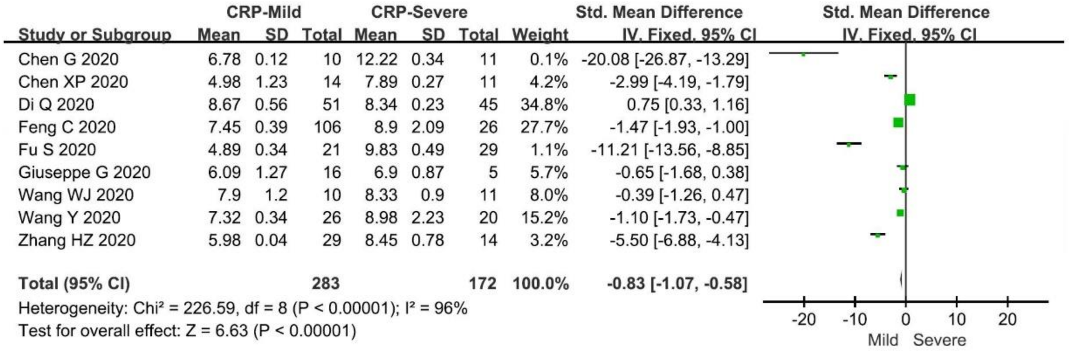

Study or Subgroup	DD-dimer-Mild Mean	SD	Total	DD-dimer-Severe Mean	SD	Total	Weight	Std. Mean Difference IV, Fixed, 95% CI
Chen G 2020	0.54	0.02	10	0.89	0.24	11	5.3%	-1.92 [-3.00, -0.85]
Di Q 2020	0.78	0.12	51	1.67	0.02	45	2.7%	-9.97 [-11.46, -8.47]
Fu S 2020	0.94	0.25	21	1.54	0.32	29	12.6%	-2.02 [-2.71, -1.32]
Yang G 2020	0.69	0.2	125	0.89	0.01	126	79.4%	-1.41 [-1.69, -1.13]
**Total (95% CI)**			**207**			**211**	**100.0%**	**-1.75 [-1.99, -1.50]**

Heterogeneity: Chi² = 122.55, df = 3 (P < 0.00001); I² = 98%
Test for overall effect: Z = 13.88 (P < 0.00001)

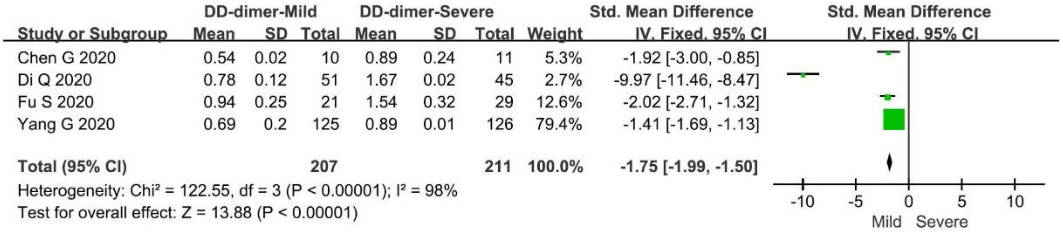

Study or Subgroup	NK cell-Mild Mean	SD	Total	NK cell-Severe Mean	SD	Total	Weight	Std. Mean Difference IV, Fixed, 95% CI
Chen G 2020	0.0356	0.001	10	0.0129	0.001	11	15.9%	21.79 [14.43, 29.15]
Wan SX 2020	0.0412	0.001	45	0.0215	0.001	18	68.5%	19.46 [15.91, 23.01]
Zhang BC 2020	0.0489	0.001	16	0.0198	0.001	16	15.5%	28.37 [20.91, 35.82]
**Total (95% CI)**			**71**			**45**	**100.0%**	**21.21 [18.28, 24.15]**

Heterogeneity: Chi² = 4.50, df = 2 (P = 0.11); I² = 56%
Test for overall effect: Z = 14.15 (P < 0.00001)

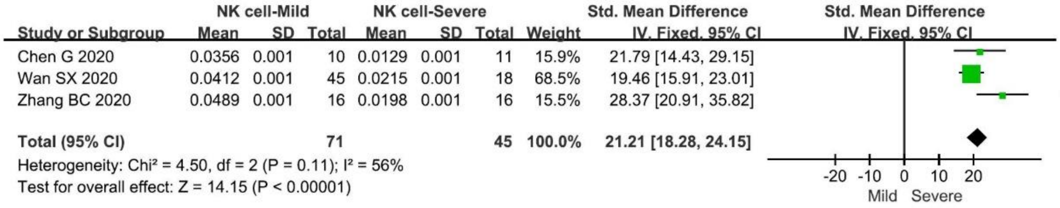

Study or Subgroup	WBC-Mild Mean	SD	Total	WBC-Severe Mean	SD	Total	Weight	Std. Mean Difference IV, Fixed, 95% CI
Chen XH 2020	9.89	1.24	48	11.78	0.98	21	16.1%	-1.60 [-2.18, -1.02]
Feng C 2020	7.24	0.28	106	8.89	1.21	26	18.2%	-2.79 [-3.34, -2.24]
Fu S 2020	11.03	3.29	21	10.25	2.78	29	17.2%	0.26 [-0.31, 0.82]
Nie SK 2020	8.46	0.89	72	10.45	0.77	25	17.3%	-2.29 [-2.85, -1.73]
Wang WJ 2020	8.08	1.24	10	14.74	2.26	11	2.6%	-3.46 [-4.90, -2.02]
Yang Y 2020	10.89	0.73	22	13.58	0.89	14	4.9%	-3.31 [-4.36, -2.26]
Zhang BC 2020	9.87	2.21	16	10.47	1.81	16	11.2%	-0.29 [-0.99, 0.41]
Zhang HZ 2020	11.08	0.73	29	9.84	2.34	14	12.4%	0.84 [0.18, 1.50]
**Total (95% CI)**			**324**			**156**	**100.0%**	**-1.30 [-1.54, -1.07]**

Heterogeneity: Chi² = 141.24, df = 7 (P < 0.00001); I² = 95%
Test for overall effect: Z = 10.91 (P < 0.00001)

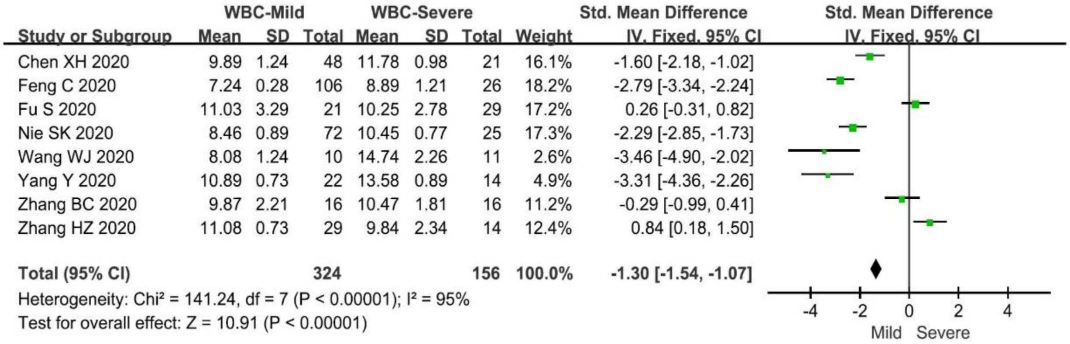

Study or Subgroup	Lymphocyte-Mild group Mean	SD	Total	Lymphocyte-Severe group Mean	SD	Total	Weight	Std. Mean Difference IV, Fixed, 95% CI
Chen G 2020	2.8	0.02	10	1.98	0.21	11	0.9%	5.15 [3.22, 7.07]
Di Q 2020	2.98	0.56	51	2.21	0.15	45	15.2%	1.81 [1.33, 2.29]
Feng C 2020	3.21	0.22	106	1.76	0.38	26	5.4%	5.58 [4.77, 6.38]
Fu S 2020	2.38	0.61	21	2.21	0.89	29	11.0%	0.21 [-0.35, 0.78]
Nie SK 2020	3.31	0.17	72	1.99	0.02	25	1.9%	8.89 [7.53, 10.25]
Wang WJ 2020	2.92	0.45	10	1.24	0.56	11	1.9%	3.16 [1.79, 4.52]
Yang G 2020	3.19	0.03	125	3.02	0.22	126	49.8%	1.08 [0.81, 1.34]
Zhang BC 2020	2.87	0.47	16	2.12	0.44	16	5.3%	1.61 [0.80, 2.42]
Zhang HZ 2020	3.01	0.81	29	3.13	0.45	14	8.6%	-0.16 [-0.80, 0.47]
**Total (95% CI)**			**440**			**303**	**100.0%**	**1.48 [1.30, 1.67]**

Heterogeneity: Chi² = 289.26, df = 8 (P < 0.00001); I² = 97%
Test for overall effect: Z = 15.56 (P < 0.00001)

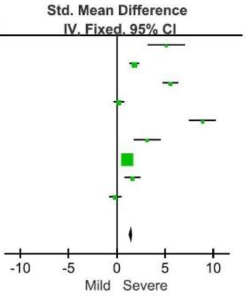

Study or Subgroup	CD3+ T cell-Mild group Mean	SD	Total	CD3+ T cell-Severe group Mean	SD	Total	Weight	Std. Mean Difference IV, Fixed, 95% CI
Chen G 2020	1,231	200	10	654	123	11	1.8%	3.38 [1.96, 4.80]
Di Q 2020	1,098	407	51	738	304	45	19.6%	0.99 [0.56, 1.41]
Fu S 2020	1,187	238	21	578	102	29	4.3%	3.48 [2.57, 4.39]
Nie SK 2020	1,315	343	72	648	383	25	12.7%	1.87 [1.34, 2.40]
Wang WJ 2020	978	45	10	367	58	11	0.2%	11.23 [7.36, 15.09]
Yang G 2020	1,098	487	125	786	342	126	54.0%	0.74 [0.48, 1.00]
Zhang BC 2020	1,435	246	16	1,032	113	16	4.6%	2.05 [1.18, 2.93]
Zhang HZ 2020	886	121	29	456	32	14	2.8%	4.15 [3.03, 5.27]
**Total (95% CI)**			**334**			**277**	**100.0%**	**1.28 [1.09, 1.47]**

Heterogeneity: Chi² = 108.54, df = 7 (P < 0.00001); I² = 94%
Test for overall effect: Z = 13.31 (P < 0.00001)

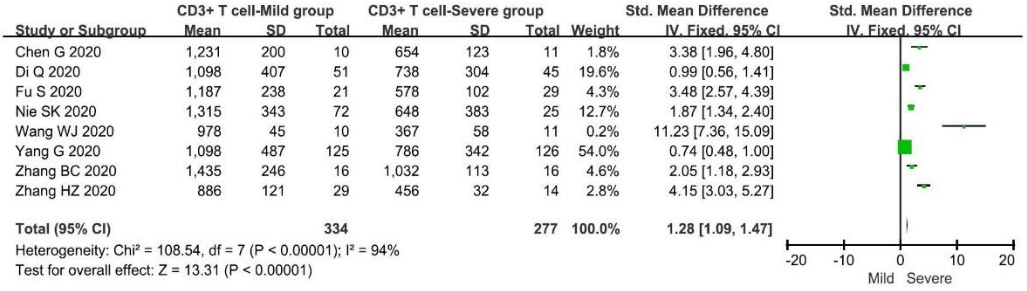

Study or Subgroup	Neutrophils-Mild Mean	SD	Total	Neutrophils-Severe Mean	SD	Total	Weight	Std. Mean Difference IV, Fixed, 95% CI
Chen G 2020	5.83	0.32	10	8.32	0.45	11	0.8%	-6.07 [-8.28, -3.86]
Di Q 2020	4.89	0.89	51	7.58	0.12	45	7.7%	-4.08 [-4.79, -3.37]
Feng C 2020	6.72	0.34	106	9.74	1.34	26	7.9%	-4.53 [-5.23, -3.83]
Fu S 2020	5.52	1.2	21	10.21	0.98	29	3.6%	-4.29 [-5.33, -3.25]
Nie SK 2020	6.23	1.24	72	9.78	0.78	25	9.7%	-3.09 [-3.72, -2.45]
Wang WJ 2020	5.56	0.12	10	8.98	1.98	11	3.0%	-2.28 [-3.43, -1.13]
Yang G 2020	7.89	0.92	125	8.9	0.67	126	53.3%	-1.25 [-1.52, -0.98]
Zhang BC 2020	7.23	1.21	16	11.21	2.01	16	4.6%	-2.34 [-3.26, -1.41]
Zhang HZ 2020	6.74	0.78	29	7.29	1.98	14	9.4%	-0.42 [-1.06, 0.23]
**Total (95% CI)**			**440**			**303**	**100.0%**	**-2.06 [-2.26, -1.86]**

Heterogeneity: Chi² = 178.38, df = 8 (P < 0.00001); I² = 96%
Test for overall effect: Z = 20.40 (P < 0.00001)

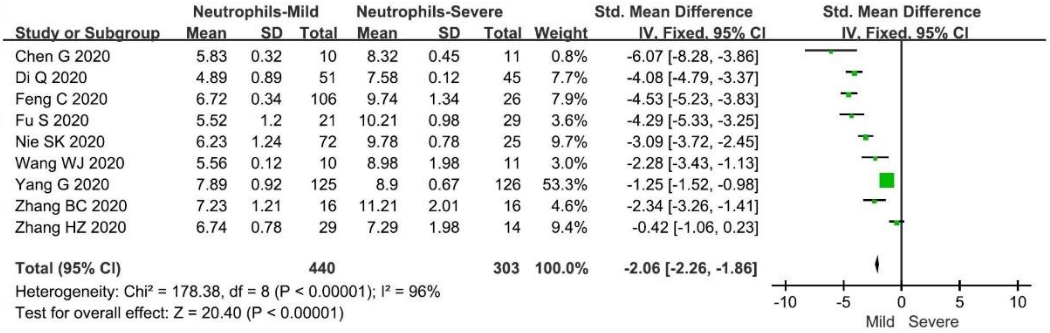

Study or Subgroup	PLT-Mild Mean	SD	Total	PLT-Severe Mean	SD	Total	Weight	Std. Mean Difference IV, Fixed, 95% CI
Chen G 2020	321	121	10	219	89	11	12.3%	0.93 [0.02, 1.84]
Di Q 2020	289	78	51	256	119	45	62.7%	0.33 [-0.07, 0.73]
Zhang HZ 2020	259	101	29	242	66	14	25.0%	0.18 [-0.46, 0.82]
**Total (95% CI)**			**90**			**70**	**100.0%**	**0.37 [0.05, 0.69]**

Heterogeneity: Chi² = 1.81, df = 2 (P = 0.40); I² = 0%
Test for overall effect: Z = 2.25 (P = 0.02)

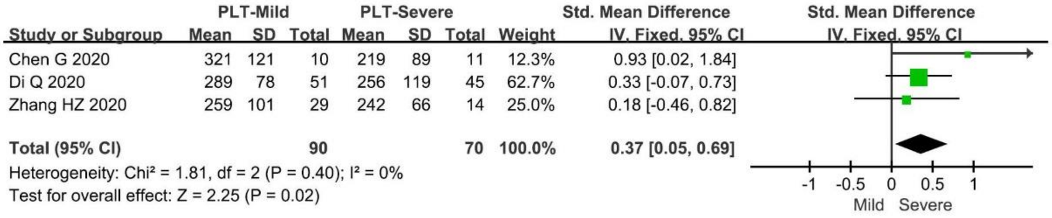

Study or Subgroup	CD4+ T cell-Mild Mean	SD	Total	CD4+ T cell-Severe Mean	SD	Total	Weight	Std. Mean Difference IV, Fixed, 95% CI
Chen G 2020	478	120	10	266	90	11	3.6%	1.93 [0.86, 3.01]
Di Q 2020	627	98	51	276	102	45	10.0%	3.49 [2.84, 4.13]
Fu S 2020	477	148	21	209	89	29	7.9%	2.25 [1.52, 2.98]
Nie SK 2020	689	178	72	372	81	25	14.4%	1.98 [1.44, 2.51]
Wang WJ 2020	742	83	10	289	121	11	1.6%	4.15 [2.52, 5.79]
Yang G 2020	672	206	125	358	89	126	45.3%	1.98 [1.67, 2.28]
Zhang BC 2020	663	289	16	478	201	16	8.0%	0.72 [0.01, 1.44]
Zhang HZ 2020	489	78	29	298	86	29	9.2%	2.30 [1.62, 2.97]
**Total (95% CI)**			**334**			**292**	**100.0%**	**2.11 [1.91, 2.31]**

Heterogeneity: Chi² = 39.37, df = 7 (P < 0.00001); I² = 82%
Test for overall effect: Z = 20.30 (P < 0.00001)

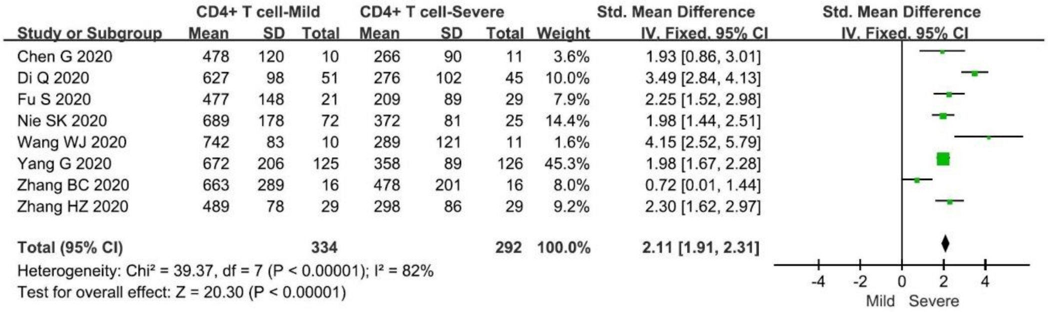

	IL-6-Mild			IL-6-Severe				Std. Mean Difference
Study or Subgroup	Mean	SD	Total	Mean	SD	Total	Weight	IV, Fixed, 95% CI
Chen G 2020	6.71	0.02	10	145.3	56.4	11	5.7%	-3.25 [-4.64, -1.86]
Chen XH 2020	4.32	0.13	48	342.4	21.2	21	0.4%	-28.86 [-33.84, -23.87]
Chen XP 2020	2.44	0.35	14	167.9	9.24	11	0.2%	-26.24 [-34.20, -18.27]
Di Q 2020	5.67	0.05	51	409.4	121.2	45	16.9%	-4.83 [-5.63, -4.03]
Feng C 2020	4.56	0.73	106	333.2	76.4	26	6.8%	-9.75 [-11.02, -8.48]
Fu S 2020	2.44	0.78	21	95.9	23.5	29	7.7%	-5.12 [-6.31, -3.94]
Giuseppe G 2020	2.44	0.42	16	89	32.2	5	2.4%	-5.62 [-7.76, -3.49]
Nie SK 2020	4.56	0.23	72	142.9	22.8	25	3.4%	-11.97 [-13.75, -10.19]
Shi YL 2020	3.9	0.61	19	323.6	102.3	25	9.5%	-4.06 [-5.13, -2.99]
Wan SX 2020	7.45	0.54	45	189.8	21.2	18	1.3%	-16.08 [-19.03, -13.13]
Wang WJ 2020	3.56	0.72	10	225.7	11.1	11	0.1%	-26.43 [-35.34, -17.52]
Wang Y 2020	7.54	0.36	22	198.4	29.09	14	1.6%	-10.37 [-13.00, -7.75]
Yang G 2020	6.37	0.46	125	287.5	67.3	126	33.1%	-5.88 [-6.45, -5.30]
Zhang BC 2020	2.87	0.09	16	76.4	18.9	16	4.5%	-5.36 [-6.93, -3.80]
Zhang HZ 2020	2.14	0.17	29	321.2	109.2	14	6.5%	-5.09 [-6.39, -3.80]
**Total (95% CI)**			**604**			**397**	**100.0%**	**-6.08 [-6.41, -5.75]**

Heterogeneity: Chi² = 298.64, df = 14 (P < 0.00001); I² = 95%
Test for overall effect: Z = 36.06 (P < 0.00001)

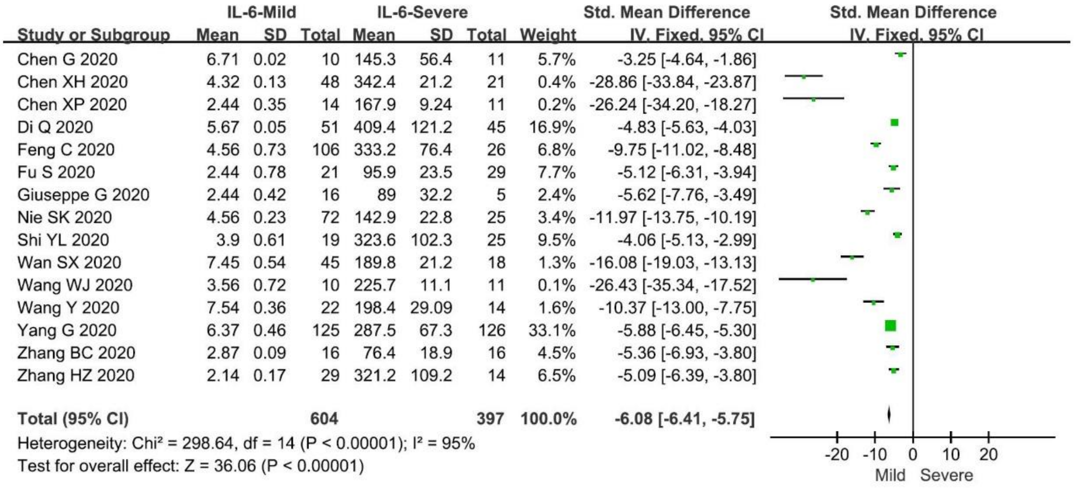

Supplement: Supplementary file 1 — Supplementary figure. [file ijmsv18p2789s1.pdf]
